# An Allele of Arabidopsis *COI1* with Hypo- and Hypermorphic Phenotypes in Plant Growth, Defence and Fertility

**DOI:** 10.1371/journal.pone.0055115

**Published:** 2013-01-30

**Authors:** Albor Dobón, Brande B. H. Wulff, Juan Vicente Canet, Patrocinio Fort, Pablo Tornero

**Affiliations:** Instituto de Biología Molecular y Celular de Plantas (IBMCP), Universidad Politécnica de Valencia (UPV)-Consejo Superior de Investigaciones Científicas (CSIC), Ciudad Politécnica de la Innovación (CPI), Valencia, Spain; Max Planck Institute for Chemical Ecology, Germany

## Abstract

Resistance to biotrophic pathogens is largely dependent on the hormone salicylic acid (SA) while jasmonic acid (JA) regulates resistance against necrotrophs. JA negatively regulates SA and is, in itself, negatively regulated by SA. A key component of the JA signal transduction pathway is its receptor, the *COI1* gene. Mutations in this gene can affect all the JA phenotypes, whereas mutations in other genes, either in JA signal transduction or in JA biosynthesis, lack this general effect. To identify components of the part of the resistance against biotrophs independent of SA, a mutagenised population of *NahG* plants (severely depleted of SA) was screened for suppression of susceptibility. The screen resulted in the identification of intragenic and extragenic suppressors, and the results presented here correspond to the characterization of one extragenic suppressor, *coi1*-40. *coi1*-40 is quite different from previously described *coi1* alleles, and it represents a strategy for enhancing resistance to biotrophs with low levels of SA, likely suppressing *NahG* by increasing the perception to the remaining SA. The phenotypes of *coi1*-40 lead us to speculate about a modular function for COI1, since we have recovered a mutation in *COI1* which has a number of JA-related phenotypes reduced while others are equal to or above wild type levels.

## Introduction

The ability of plants to prevent pathogen colonization relies on a complex network of genes and phytohormones. Salicylic acid (SA) is a well known hormone essential for activating plant basal defence responses, particularly against biotrophic pathogens (reviewed by [1]). An imbalance in basal levels of SA can dramatically alter plant resistance. For instance, *Arabidopsis thaliana* (Arabidopsis) plants with high levels of SA are more resistant to pathogens such as the bacteria *Pseudomonas syringae* pv. *tomato* isolate DC3000 (*Pto*) [2], while plants with lower SA levels are less resistant to *Pto* and other pathogens [3]. Furthermore, it has been shown that transgenic plants expressing the salicylate hydroxylase gene from *Pseudomonas putida* (*NahG*) can rapidly degrade SA [3] and are therefore more susceptible to biotrophic pathogens [4] such as *Pto* [5]. Once activated, SA resistance triggers a number of defence or pathogenesis-related genes including *PR1*. This gene is widely used as a marker for biotic stress and is required for various types of resistance, including Systemic Acquired Resistance (SAR). SAR acts to protect systemic leaves following earlier localized pathogen inoculation [6]. Considering the array of resistance responses to biotrophs, there is evidence for part of the resistance response being independent of SA [7].

A phytohormone with an intricate relationship with SA is jasmonic acid (JA). JA is required for a wide range of plant functions, from pollen maturation to activating defence responses against necrotrophic pathogens (reviewed by [8]). It also plays a minor role in activating defence responses against biotrophs, since exogenous application of jasmonates, i.e. methyl esther jasmonate (MeJA), can trigger defence against *Pto*, a response that appears dependent on activation of *NPR1* [9]. JA is also important for inducing systemic induced susceptibility (SIS, [10]), which, in contrast to SAR, induces susceptibility in systemic leaves. As with SA, there are several genes that play a significant role in JA signal transduction, but only its receptor, *COI1*, is absolutely required for inducing all related phenotypes. SA and JA have been shown to negatively regulate each other, although there are examples of synergistic effects (reviewed in [11]).

This study aims to investigate resistance responses that are independent of SA. Although there are no viable biosynthetic mutants that are completely deficient of SA [12], *NahG* plants are severely depleted in SA [3]. Using a mutagenised *NahG* population and screening for suppressors of *NahG* susceptibility, we aimed to identify and characterize parts of the resistance response that would normally be masked by the abundance of SA. The results presented here show that part of the SA-independent defence response is dependent on JA perception. In addition, we show that an allele of *COI1* displays a number of JA-related phenotypes reduced while others are equal to or above wild type levels.

## Results

### Design and implementation of a *NahG* suppressor screen

In a previous screen for loss of resistance to *Pto*, one of us (P.T.) recovered a promising Arabidopsis mutant, *lra5* [13]. Further characterization showed that *lra5* was in fact a stray *NahG* plant that contaminated the screen (data not shown). The *NahG_CW_* line was found to have some islands in the genome from the accession Ws-0 (data not shown), hence the name. Then, it was backcrossed five times with the accession Col-0. The transgene was inserted between the genes At2g46970 and At2g46980, and there were no differences in susceptibility to *Pto* with other Col-0 or Ws-0 *NahG* lines (data not shown).

We took advantage of the detailed characterization of this line to elucidate how the SA-dependent and independent branches of the resistance response interact, by using *NahG_CW_* to screen for mutations that suppress the susceptibility to *Pto*. Before embarking on the *NahG_CW_* suppressor screen, the conditions were optimized using several Arabidopsis mutants with an enhanced resistance against *Pto* [1]. We generated double mutants between *NahG_CW_* and *cpr1* [14], *cpr5* [15], *dnd1* [16], and *lsd1* [17]. With these plants, we fine-tuned a medium throughput screening protocol that would detect suppressors of *NahG* susceptibility. Figure S1 shows the proof of concept after optimizing the inoculations. With two inoculations of *Pto*, wild-type plants of the ecotypes Col-0, Ws-0 and La*er*-0 can overcome the pathogen and grow almost unaffected, while *NahG_CW_* plants died or were severely affected. The enhanced disease resistance mutants in combination with *NahG_CW_* produced a small but detectable suppression of *NahG*, but in the case of *cpr5 NahG_CW_*, there is a strong suppression of the susceptibility (Figure S1, also described by [15]).

From 60 independent M2 families, 89 candidates were recovered and 40 selected for further characterization. These 40 putative mutants were crossed with Col-0, and their F2 progeny were inoculated with *Pto*. We identified 12 intragenic and 28 extragenic suppressors. Three of the intragenic suppressors were selected and characterized further to confirm that they were allelic and less susceptible to *Pto* than the parental line *NahG* (Figure S2A and S2B, respectively).

An extragenic suppressor, *coi1*-40, was selected for further characterization. In our conditions *coi1-*40 was not different to wild type in all the gross morphological phenotype (data not shown). This mutant was shown to contain a single nuclear mutation, which was recessive and effectively suppressed the susceptibility of *NahG*. *coi1*-40 was mapped to Chromosome II, between the markers C2-12916335 and BIO2-18012804 (an interval of 5.1 Mb).

### Response to pathogens of *coi1-*40

Since *NahG* plants accumulate a very low but discernable level of SA [18], it was important to ascertain if the suppression of susceptibility in *coi1*-40 plants was related to alterations in SA levels or other mechanisms. The steady state levels of SA in *coi1*-40 were similar to Col-0, and *coi1*-40 *NahG_CW_* accumulated similar levels compared to *NahG_CW_* (Figure S3).

**Figure 1:**
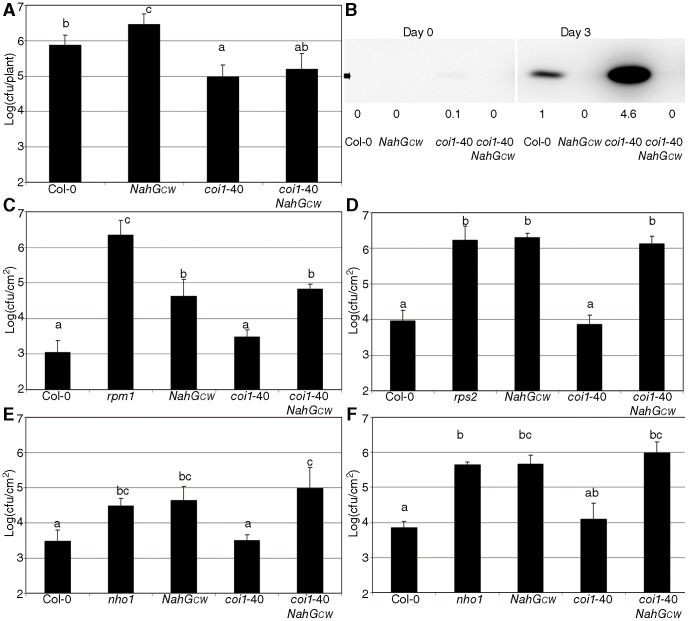
Characterization of resistance to biotrophs in *coi1*-40. (A) Growth of *Pto* in the suppressor. Plants of the indicated genotypes were spray-inoculated with *Pseudomonas syringae* pv. *tomato* isolate DC3000 (*Pto*) at an OD_600_ of 0.1 when they were 18 days old. (B) PR1 Western blot of the indicated genotypes at day zero and three days post inoculation with *Pto*, inoculated as described in (A). The arrow indicates the position of PR1 (14 kDa). The same membrane was probed with anti-RuBisCO, as a loading and transferring control. The signal produced by anti-PR1 was quantified and normalized against the control of anti-RuBisCO. The data is shown in arbitrary units, where the amount in Col-0 inoculated with *Pto* is equal to one. (C) Growth of *Pto*(*avrRpm1*) in the suppressor. *rpm1* is included as a control. (D) Growth of *Pto*(*avrRpt2*) in the suppressor. *rps2* is added as a control. (E) Growth of *Pseudomonas syringae* pv. *phaseolicola* isolate NPS3121 in the suppressor. (F) Growth of *Pseudomonas syringae* pv. *tabaci* in the suppressor. In both (E) and (F) *nho1* is used as a control. In the panels (C) to (F), 28 day-old plants were inoculated by hand infiltration with bacterial suspension at an OD_600_ of 2×10E-4, since it is the best way to characterize these resistances. The data represent the average and the standard deviation of three measurements, and in all the figures, the experiments were repeated three times with similar results. The letters above the bars indicate different homogeneous groups with statistically significant differences (Fisher's LSD Test, P<0.05).

Identification of the suppressor mutants was based on visual inspection of disease symptoms. It is possible, however, that the reduced macroscopic disease symptoms did not reflect reduced pathogen growth. Therefore, more accurate measurements of *Pto* growth were performed. These measurements confirmed that *coi1*-40 is able to suppress the growth of *Pto* in a *NahG* background (Figure 1A). In fact, the single mutant was more resistant than Col-0, even in the *NahG* background, demonstrating that *coi1-*40 has a heightened basal resistance. The status of resistance can also be evaluated by the levels of the protein PR1 [19]. Upon *Pto* inoculation PR1 was strongly induced in *coi1*-40 compared to the Col-0 control, however, in *coi1*-40 *NahG_CW_* no induction was evident (Figure 1B). The same membranes were probed with anti-RuBisCO as an internal control (Figure 1B).

*NahG* is not only susceptible to compatible pathogens like *Pto*, but also to some incompatible and non-host pathogens [20]. Figure 1C–D displays the behaviour of *coi1*-40 when inoculated with *Pto(avrRpm1)* [21] or *Pto(avrRpt2)* [22]. The presence of the *avrRpm1* or *avrRpt2* effectors converts *Pto* into an incompatible pathogen in the presence of the resistance genes *RPM1* [23] and *RPS2* [24], respectively, and *coi1*-40 did not suppress the susceptibility to either effector (Figure 1C and D). Analogous results were obtained when the genotypes were inoculated with the non-host pathogens *Pseudomonas phaseolicola* (*Pph*) and *Pseudomonas tabaci* (*Ptab*) (Figure 1E–F).

**Figure 2:**
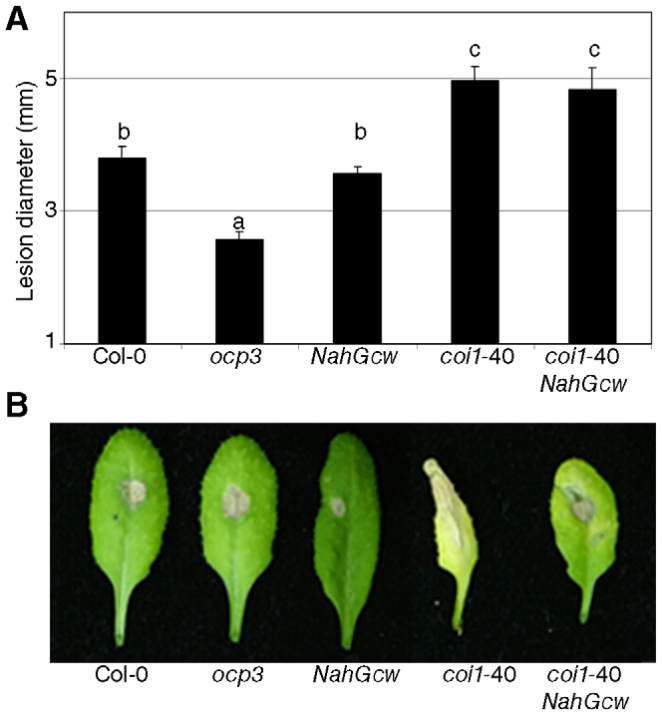
Resistance against necrotrophs in *coi1*-40. *coi1*-40 and its controls were inoculated with *Plectosphaerella cucumerina* by depositing a 6 µL drop of 5×10E6 spores/mL on a leaf, in 28-day-old plants. (A) Diameter of the lesion one week after inoculation. *ocp3* is included as a control. The data represent the average and the standard error of 40 leaves. (B) Images of representative leaves two weeks after inoculation. The genotypes are shown in the same order as in (A).

As mentioned previously, pathogen resistance can be activated independently of SA signalling. To assess the resistance of *coi1*-40 against necrotrophic pathogens, which is dependent on JA signalling [25], the mutants were inoculated with *Plectosphaerella cucumerina* (Figure 2). *coi1*-40 showed a marked increase in susceptibility to *P. cucumerina* one week after inoculation. Figure 2B shows the leaves of an experiment when the sampling is done two weeks after inoculation; note that *coi1*-40 *NahG_CW_* was slightly less susceptible than *coi1*-40 alone. *ocp3*, a mutant more resistant to *P. cucumerina*, is included as a control [26]. *OCP3* is a homeodomain transcription factor, and a mutation in this gene renders plants more resistant to necrotrophic pathogens without affecting the resistance to biotrophs [26].

### JA defence-related phenotypes of *coi1*-40

**Figure 3:**
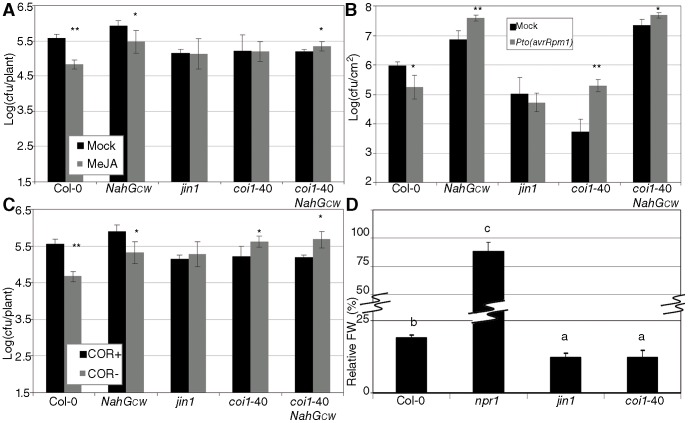
Pathogen resistance phenotypes of *coi1*-40 related to JA. *coi1*-40 and its controls were tested for: (A) Methyl jasmonate (MeJA) induced resistance. 17-day-old-plants were treated with either 100 µM MeJA (with 0.1% DMSO and 0.02% Silwet L-77) or a mock solution. One day later, *Pto* was inoculated and its growth measured as in Figure 1A. (B) Systemic Acquired Resistance. Three leaves of 28-day-old plants were hand infiltrated with either *Pto(avrRpm1)* or a mock solution. Two days later, *Pto* was inoculated and its growth in systemic leaves measured as described (see Methods). (C) Coronatine as a virulence factor. Bacteria with coronatine (*Pto*, COR+) or without coronatine (*Pto(cfa^−^*), COR−) were inoculated and their growth measured as in Figure 1A. (D) Negative crosstalk with SA. Plants of the indicated genotypes were treated four times with either 350 µM benzothiadiazole (BTH) or a mock solution, and their weight was recorded when 21 days old. The ratio between BTH treated and mock treated is shown as a percentage, with *npr1* included as control. *jin1* is included in all the panels as a control of no response to JA. Asterisks indicate statistically significant differences from the mock treatment (P<0.05 one asterisks, P<0.01 two) using the Student's t-test (two-tails).

Given the enhanced susceptibility of *coi1-*40 to *P. cucumerina* and that resistance against necrotrophs is dependent on JA, we reasoned that it would be interesting to test a series of JA related phenotypes in *coi1*-40. Figure 3A shows the resistance induced by MeJA when *Pto* is inoculated one day later [27]. Both Col-0 and *NahG_CW_* respond to MeJA with a small but reproducible reduction in the levels of *Pto*, compared to the negative control *jin1* (*Jasmonate Insensitive 1*, [28]). In these conditions, no resistance response was evident for *coi1*-40 and *coi1*-40 *NahG_CW_* in response to MeJA. SAR has been shown to be dependent on JA, [29] and in accordance wild-type Col-0 plants displayed SAR. However, *NahG_CW_* and *jin1* (Figure 3B), *coi1*-40 and *coi1*-40 *NahG_CW_* had no SAR and *Pto* grew better in SAR conditions, especially for *coi1*-40 plants. This phenotype has been called SIS [10], and it is a systemic effect of coronatine. Some isolates like *Pto* are able to produce this chemical, a molecular mimic of the iso-leucine conjugate of jasmonic acid (JA-Ile, [30]), that functions as a virulence factor [31]. We inoculated *coi1*-40 and *coi1*-40 *NahG_CW_* and their controls with a *Pto* strain that lacks coronatine (*Pto(cfa^−^)*, [31]), and a wild-type *Pto* strain (Figure 3C). *Pto(cfa^−^)* had a reduced growth in wild-type plants, compared to *Pto*, while in *coi1*-40 and *coi1*-40 *NahG_CW_* there were no detectable differences, or the differences were opposite to those on wild-type plants (Figure 3C).

One of the hallmarks of JA signalling in defence is the negative crosstalk with SA signal transduction [32]. Therefore, we measured the perception of benzothiadiazole (BTH, an analogue of SA, [33]) to check the status of crosstalk in *coi1*-40 plants. Wild-type, *jin1* and *coi1*-40 plants had a considerable reduction in fresh weight, when compared to *npr1* and wild type plants (Figure 3D). The increase in sensitivity to BTH for these mutants is consistent with less negative crosstalk from JA to SA signalling pathways.

### JA growth-related phenotypes of *coi1*-40

**Figure 4:**
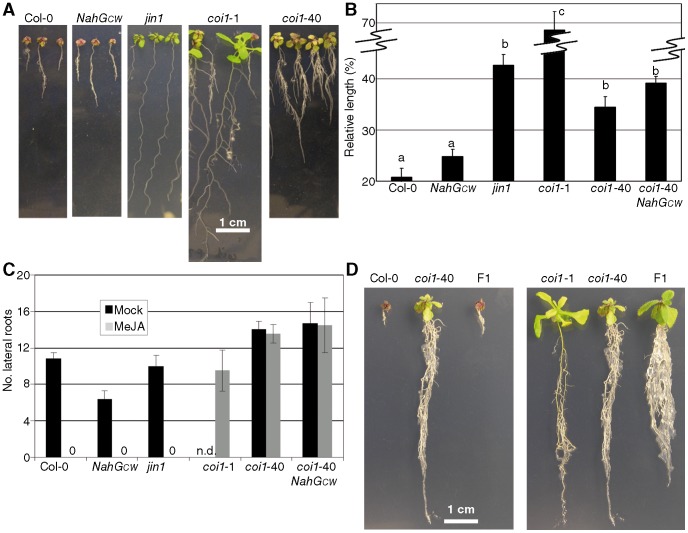
Response of *coi1*-40 to JA *in vitro*. *coi1*-40 and its controls were tested for: (A) Phenotype in plates. The indicated genotypes were grown in plates with Johnson's Media [67] supplemented with 50 µM MeJA. The pictures were taken 20 days after germination with the same settings and in the same experiment. In plates without MeJA the plants were the same size (data not shown). (B) Length of primary root. The plants were grown as described in (A), with and without 50 µM MeJA. At 10 days old, the lengths of the roots were measured in both conditions, and their ratio (MeJA treated divided by mock treated) expressed as a percentage. (C) Lateral roots. The plants were grown as described in (A), with and without 50 µM MeJA. At 14 days old, the number of lateral roots longer than 0.2 mm was counted in both conditions with the help of a magnifying glass. Note that in some genotypes like Col-0, the root does not grow in MeJA and therefore it is not possible to count lateral roots (marked as “0” in the figure). Since *coi1*-1 is not fertile, the number of lateral roots without MeJA was not counted (marked as not determined -n.d.- in the figure). (D) Phenotype of F1s between *coi1*-40 and Col-0 and between *coi1*-40 and *coi1*-1. The plants were grown as described in A, with 50 µM MeJA. The pictures were taken when the plants were 20 days old.

To determine whether perception of JA in *coi1*-40 was affected during development, we studied the phenotypes of *coi1*-40 alongside *coi1*-1. The receptor of JA, COI1 (*Coronatine Insensitive 1*, [34]), is an F-box protein and it is a key regulator of JA signalling, therefore null alleles are insensitive to JA. The plants were grown *in vitro* in the presence and absence of MeJA. In the absence of MeJA, all plants were comparable, while in the presence of MeJA, several phenotypes were evident (Figure 4A). Firstly, the aerial region of *coi1*-40 was larger than the wild-type control, although not as large as *coi1-*1. Secondly, the relative length of the primary root, in *coi1*-40 was intermediate between Col-0 and *coi1*-1 (Figure 4B). Thirdly, there was a profusion of secondary root growth (branching) in *coi1*-40.

JA has been reported to induce root branching [35], therefore, to quantify this phenotype, we counted the number of lateral roots in 14 day-old seedlings grown in medium in the presence or absence of 50 µM MeJA. We found that *coi1*-40 had more lateral roots than *coi1*-1 when grown in the presence of MeJA, or Col-0 in mock conditions (Figure 4C). We could not sample *coi1*-1 in mock treatment at 10–14 days old, due to its lack of phenotype at this stage in a segregating population. Since JA-induced secondary branching increases the length of the root system we used total root dry weight as a measure of the size of the entire root system. This analysis revealed that the relative weight of *coi1*-40 roots was similar to *coi1*-1 (Figure S4).

**Figure 5:**
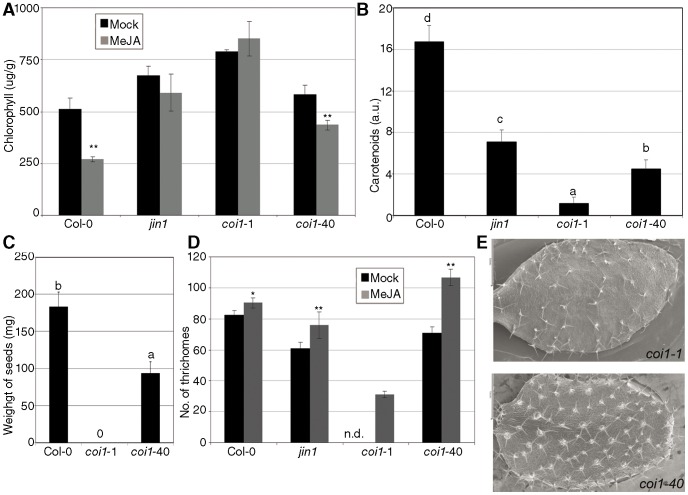
Allele specific phenotypes of *coi1*-40. *coi1*-40 and its controls were tested for: (A) Senescence induced by JA. The indicated genotypes were grown in soil, and mature leaves from six-week-old plants were cut and floated on water with or without 100 µM MeJA. The amount of chlorophyll (in µg/g fresh weight) was measured after four days of darkness, with three groups of leaves of *c.* 1 g each. Previous to the sampling, *coi1*-1 plants were selected by PCR markers from a segregating population. (B) Carotenoids. 14-day-old seedlings, grown in 50 µM MeJA plates, were incubated in acetic methanol during 18 hours and the absorption of the extracts was measured. (C) Fertility. The total average seed set of eight plants grown in long day conditions. (D) Trichomes. Plants were grown in media with or without 10 µM MeJA, and when the fifth true leaf emerged, the number of trichomes was counted with the help of a magnifying glass. Since *coi1*-1 is not fertile, the number of trichomes without MeJA cannot be counted at this stage. (E) Detail of the distribution of trichomes in both *coi1* alleles. Plants growing in MeJA plates, as indicated in (D) were visualized with a scanning electron microscope. The length of the bar (left of the picture) is 1 mm.

Senescence in leaves is accelerated by JA when incubated in the dark [36]. Figure 5A shows that, in wild-type plants, MeJA promoted a loss of chlorophyll. *jin1* and *coi1*-1 did not show this effect, while *coi1*-40 was intermediate between *coi1*-1 and Col-0.

The synthesis and accumulation of carotenoids is activated by many abiotic and biotic signals, including JA. In addition, this biosynthesis is modulated by *COI1* [37]. By growing plants in MeJA-containing media and measuring the levels of carotenoids (Figure 5B), we found that *coi1*-40 was intermediate between *coi1*-1 and Col-0. One of the main characteristics of *coi1*-1 is its lack of fertility. For this phenotype, *coi1*-40 was also found to be intermediate between wild-type and *coi1*-1 (Figure 5C).

The number of trichomes is another developmental phenotype partially dependent on JA, [38]. We found that the number of trichomes for *coi1*-40 was enhanced in the presence of MeJA (Figure 5D), and that the morphology was strikingly different to wild-type plants (Figure 5E, S6).

### *coi1*-40 is an allele of *coi1*

*coi1*-40 and *coi1*-1 displayed quite different JA-dependent phenotypes, such as *coi1*-40 being fertile. However, both mutants shared certain phenotypes such as dry weight of the root system in MeJA, and *coi1*-40 mapped to an interval that contains *COI1* (At2g39940). In order to check for complementation, the F1 plants from a cross between *coi1*-1 and *coi1*-40 or Col-0 and *coi1*-40 were obtained and grown in MeJA-containing media (Figure 4D). The F1 from the *coi1*-1 by *coi1*-40 cross showed identical phenotypes to *coi1*-40, when root length, number of lateral roots, and number of trichomes were assessed. This suggests that *coi1*-40 is an allele of *COI1* (Figure S7) or that both mutations are in different genes that interact genetically and could give rise to non-allelic, non-complementation. However, in the F2 all plants were resistant to MeJA, and 3 in every 4 were similar to *coi1*-40, while 1 in every 4 was similar to *coi1*-1 (data not shown). Therefore, both mutations are allelic, and *coi1*-40 has allele-specific phenotypes. The F1 plants from the cross between Col-0 and *coi1*-40 displayed a wild type phenotype when grown in the presence of MeJA (Figure 4D) and the F2 segregated 3∶1 (Col-0 to *coi1*-40), indicating that *coi1*-40 is a recessive mutation. Sequencing of *COI1* in *coi1*-40 revealed a single canonical EMS mutation in the gene, changing residue 22 from glutamic acid to lysine, indicating again that *coi1*-40 is allelic to *coi1*-1 (Figure S5).

The lateral root, length of the main root, and trichome phenotypes were dominant in *coi1*-40 with respect to *coi1*-1, but recessive with respect to wild type *COI1* (Figure 4D and Figure S7). Once the mutation was identified, 60 F2 from *coi1*-1×*coi1*-40 and 60 F2 from Col-0 × *coi1*-40 crosses were analyzed with a molecular marker and the lateral root phenotype was found to cosegregate with the *coi1*-40 marker in both populations (data not shown). The trichome phenotype was also visually observed as cosegregating with the molecular marker in 70 F2s from the cross *coi1*-1×*coi1*-40, due to the low level of trichomes in *coi1*-1 (Figure 5D and E).

### JA-induced expression of COI1-dependent genes in *coi1-*40

**Figure 6:**
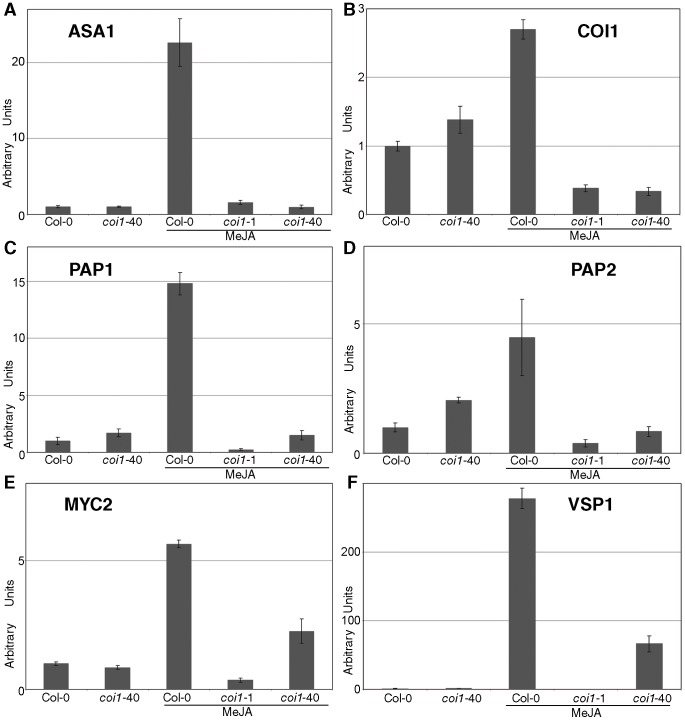
Allele specific molecular phenotypes of *coi1*-40. Col-0, *coi1*-1, and *coi1*-40 plants were grown both in mock and 50 µM MeJA plates. RNA was extracted 10 days after germination, and transcript levels for the following genes were measured by RT-qPCR: (A) *ASA1*; (B) *COI1*; (C) *PAP1*; (D) *PAP2*; (E) *MYC2*; (F) *VSP1*. The levels of expression are normalized to three reference genes and to the level of Col-0 in mock.

The differences in some of the JA-dependent phenotypes shown by Col-0 and the *coi1-*1 and *coi1-*40 mutants should mirror a molecular footprint. Therefore, we analyzed the effect of MeJA on the expression of six important genes involved in JA-related phenotypes (Figure 6). The *ASA1* gene (*Anthranilate Synthase α1*, [35]) may modulate auxin biosynthesis in response to JA thus regulating lateral root formation. We analyzed the expression of this gene to address whether the lateral root phenotype of *coi1-40* was dependent of this node (Figure 6A). No significant differences were observed; hence the phenotype of *coi1-40* might be independent of this auxin pathway. The expression of *COI1* was also examined and found to be identical in the two *coi1* alleles (Figure 6B). *PAP1* and *PAP2* (*Production of Anthocyanin Pigment 1* and *2*, [39]) are two *MYB* genes that mediate the JA-dependent anthocyanin biosynthesis. It has been reported that the induction of both genes by JA is also dependent of *COI1* [37]. In response to MeJA, *coi1*-40 maintained *PAP1* (Figure 6C) and displayed reduced levels of *PAP2* (Figure 6D). However, both displayed higher expression levels than in *coi1*-1. The expression of two further genes was also assessed, *MYC2* and *VSP1*. *MYC2* is a central transcription factor for most JA-induced responses and *VSP1* (*Vegetative Storage Protein 1*, [40]) is a specific marker induced by JA. In both we found a stronger difference between these alleles. There is a certain amount of JA signalling that goes through *coi1*-40 inducing the expression of *MYC2* (Figure 6E) and *VSP1* (Figure 6F) to considerable levels, which could explain some of the phenotype differences between *coi1*-1 and *coi1*-40.

**Figure 7:**
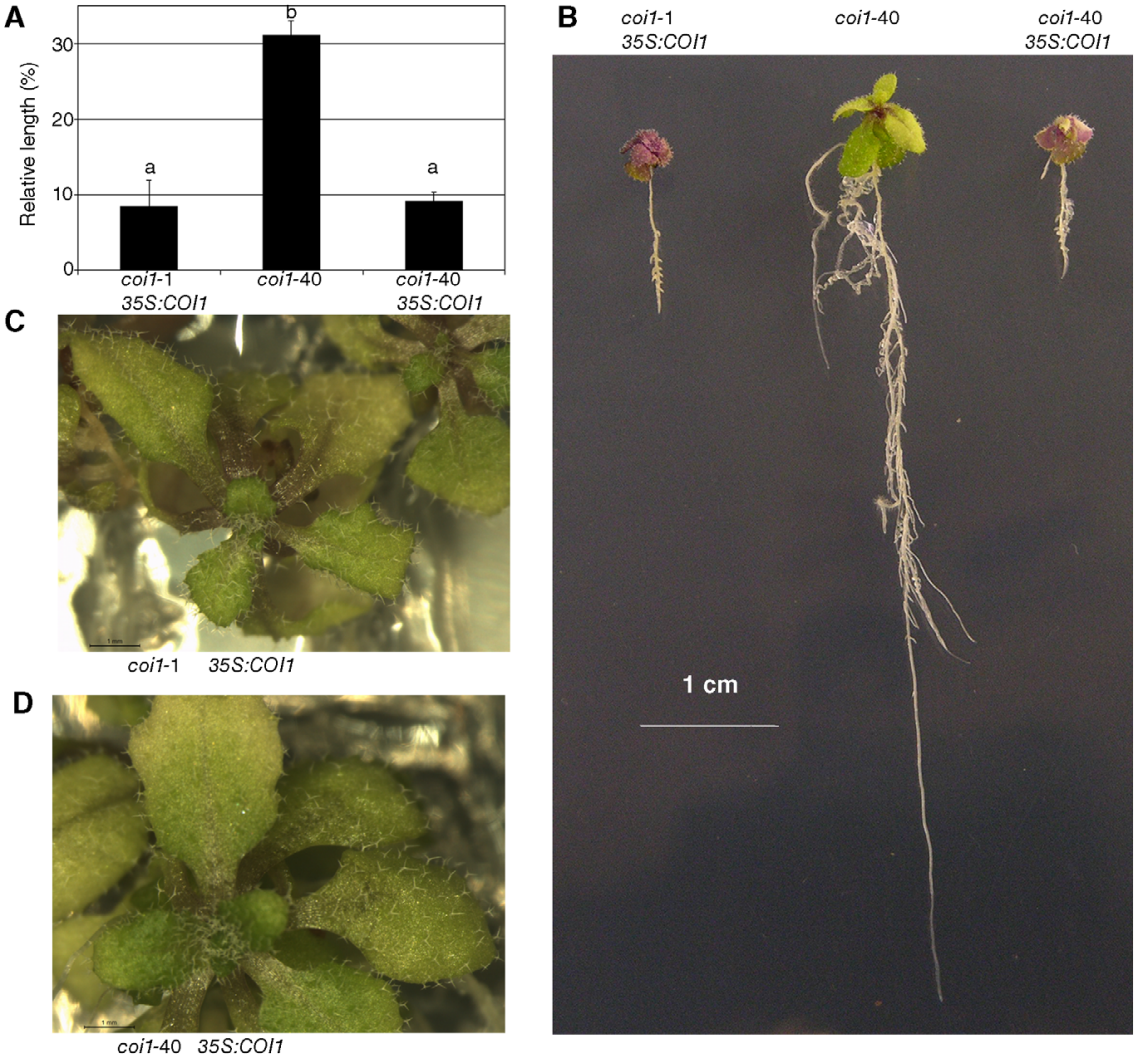
Complementation of *coi1*-40. Plants of *coi1-*40, *coi1-*1 *35S:COI1* and *35S:COI1* in *coi1-*40 background were grown in plates supplemented with and without MeJA as is described in Figure 4. (A) Length of primary root. The plants were grown with and without 50 µM MeJA. At 10 days old, the lengths of the roots were measured in both conditions, and their ratio (MeJA treated divided by mock treated) expressed as a percentage. (B) Lateral root phenotype. Picture showing the phenotype of the three lines, *coi1-*40, *coi1-*1 *35S:COI1*, *coi1-*40 *35S:COI* 20 days post germination. Both lines, *coi1-*1 *35S:COI1* and *coi1-*40 *35S:COI* show similar phenotype and opposite to that shown in the control *coi1*-40. (C) and (D) Trichome phenotype. Plants were grown in media with 10 µM MeJA. No difference in the number of trichomes was found between *coi1-*1 *35S:COI1* (C) and *coi1-*40 *35S:COI* (D). The picture shows the plants when the fifth true leaf emerged.

We complemented the *coi1*-40 mutation by crossing it with a transgenic line that express COI1-Flag under the control of the promoter 35S [41]. The *coi1-40 35S:COI1-Flag* behaved as the control *coi1*-1 *35S:COI1-Flag* homozygous line for relative length of the root (Figure 7A and B) and number of trichomes (Figure 7C and 7D) demonstrating that these phenotypes are caused by the mutation in *coi1-*40.

## Discussion

### A *NahG* extragenic suppressor

Resistance against biotrophs in plants depends largely on SA accumulation [42]. As *NahG* plants have reduced levels of SA, this provides a background where mutations that enhance the resistance in an SA independent manner are easily recognizable. Here we report the screening of a *NahG* mutagenised line, which resulted in the identification of intragenic (mutations in the *NahG* transgene itself), and extragenic mutations. An extragenic mutant, *coi1*-40, was fully characterized, and shown to be more resistant against biotrophic pathogens than the parental lines both in the presence or absence of *NahG* (Figure 1A).

*coi1*-40 is an interesting and informative allele by itself, as described below. However, our initial objective was to analyze the interaction between SA-dependent and independent branches of the resistance response. *coi1*-40 was found in our screen because it suppresses the susceptibility of *NahG* (Figure 1A). This suppression is produced by two mechanisms. First, the insensibility of the mutant to coronatine depletes the advantage that this chemical gives to the pathogen [31]. Second, since the steady-state level of PR1 protein in *coi1*-40 is almost undetectable but is strongly induced upon *Pto* inoculation, (Figure 1B), we speculate that *coi1*-40 increases the sensitivity to pathogen signals that trigger resistance. As *coi1*-40 was identified as an allele of *COI1*, mutating *coi1* would potentially increase sensitivity to SA, since in wild type plants there is negative crosstalk between JA and SA [43]. The same increased sensitivity is shown with respect to BTH (Figure 3D).

Therefore, one of the mechanisms of *coi1*-40 suppressing *NahG* susceptibility is SA-independent (coronatine is no longer a virulence factor), but the other mechanism is SA-dependent (enhanced perception of SA). The two incompatible (Figure 1C and 1D) and the two non-host interactions (Figure 1E and 1F) were not affected by *coi1*-40. Therefore, increased sensitivity to SA and lack of coronatine recognition conferred by *coi1*-40 mutants has no effect on these interactions although it showed enhanced basal resistance. On the other hand, the response to *P. cucumerina* in *coi1*-40 was severely compromised (Figure 2) pointing to an impairment in the JA-disease resistance against necrotrophic pathogens. Interestingly, the response to *P. cucumerina* also shows the negative regulation from SA to JA. Thus, when the leaves were sampled two weeks after inoculation (Figure 2B), *coi1*-40 *NahG* were slightly less susceptible than *coi1*-40, likely due to the reduced perception of SA in *NahG*, that leads to the increased perception of JA by a weak allele of *COI1*.

### *coi1-*40 differentiates phenotypes related to JA in roots

*coi1*-40 shares several phenotypes with other JA mutants such as *jin1* or *coi1*-1. Thus, like *coi1*-1 [34], *coi1*-40 is fully susceptible to *P. cucumerina* (Figure 2). When exogenously applied, MeJA is able to induce a small resistance against subsequent *Pto* infections [27]. *coi1*-40 does not trigger this resistance (Figure 3A), nor SAR (Figure 3B, [44]), although it has an enhanced perception of SA and its analogues (Figure 3D, [45]).

There are certain differences between the phenotypes induced by the *coi1*-40 and *coi1*-1 alleles (Table 1). Four of these phenotypes indicate that *coi1*-40 is a hypomorph, i.e. intermediate between Col-0 and the *coi1*-1 null mutant. These unrelated phenotypes include the relative length of roots growing in MeJA plates, the senescence induced by JA, the production of carotenoids induced by JA, and the fertility. While two are produced by exogenous application of MeJA, two are responding to endogenous levels. While two are on sterile plates, two are in soil. From the mentioned phenotypes, it is tempting to merely assign a weak or leaky character to *coi1*-40, however, there are three phenotypes in which *coi1*-40 behaves as a hypermorph; lateral root growth, trichome development and SIS.

**Table 1:** Summary of the phenotypes that differentiate the *coi1-*40 and *coi1*-1 alleles.

	Genotypes			
Phenotypes	Col-0	*coi1-*40	*coi1-*40×*coi1-*1 F1	*coi1-*1
Root lenght^1^	+	++	++	+++
Senescence^2^	+++	++	n.d.	+
Carotenoids^1^	+++	++	n.d.	+
Fertility	+++	++	++^3^	0
Lateral roots^1^	+	+++	++++	++
Trichomes^1^	++	+++	+++	+
SIS	+	++	n.d.	0 [10]

1 Measured in plants grown on MeJA supplemented plates;

2 Senescence induced by MeJA.

3 Amount of seeds estimated. n.d.: not determined.

The development of lateral roots is orchestrated by the distribution of auxins (basipetal in the root and acropetal in the leaf [46]). Auxin application stimulates the formation of lateral roots [47], while inhibitors of auxins prevent the formation of them [46]. Therefore, the increased number of lateral roots in *coi1*-40 may be brought about by an increase in production or perception of auxins. The gene *ASA1* (*ANTHRANILATE SYNTHASE α1*) is an auxin biosynthesis gene responsible for lateral root formation in the presence of JA [35]. Expression analysis has shown that the expression of *ASA1* is *COI1* dependent (Figure 6A, [35]). Therefore, ASA1 could act to integrate JA and auxin signalling. While the line of argument for auxins being involved in the formation of lateral roots in *coi1*-40 is appealing, this could not be verified experimentally. Thus, *ASA1* was not induced by MeJA in *coi1*-40 (Figure 6A), nor was there any apparent phenotype of the *coi1*-40 mutant in 2,4-D plates (data not shown).

### *coi1-*40 produces more trichomes in response to JA than the wild type

Arabidopsis responds to wounding or MeJA applications by increasing the number of trichomes in the newly formed leaves [48]. Surprisingly, not all mutants in JA signalling are defective in trichome response to JA [38]. In *coi1*-40 this response to JA is hypermorphic (Figure 5D and E). The production of trichomes in Arabidopsis involves a complex genetic model, including *Glabra3* (*GL3*, [49]), among other genes. JA induces the expression of *GL3*, setting in motion the formation of trichomes [38]. In *coi1*-40, the levels of *GL3* are not altered upon MeJA treatment (data not shown).

### SIS

*Pto* grows better in *coi1*-40 plants where SAR has been triggered, which could indicate that, like other mutants in JA signalling, *coi1*-40 is negatively affected in SAR and displays a hypermorphic SIS [10]. Although the initial observation of SIS was obtained from incubation with a virulent strain, there is evidence that an avirulent strain can also trigger SIS [10]. While in a wild type plant this effect would be overcome by SAR, *coi1*-40 allows separating these two opposing tendencies, favouring SIS (Figure 3B).

### Behaviour of F1s

The lateral root and trichome phenotypes are dominant with respect to *coi1*-1; one copy of *coi1*-40 increases both the number of lateral roots and trichomes if the other allele is *coi1*-1 (Figure 4D and Figure S7). This fact implies that any explanation of the mentioned phenotypes by secondary EMS mutations in *NahG_CW_* is highly unlikely, since all the phenotypes are dependent on the locus *COI1*. In addition, these phenotypes cosegregate perfectly in a dominant fashion with a molecular marker for *coi1*-40 in an F2 segregating family of *coi1*-1×*coi1*-40. Similarly, in the cross *coi1*-40×Col-0, the lateral root phenotype cosegregates perfectly in a recessive fashion with the same marker. In contrast, the lateral root phenotype was not seen in F2 populations from *NahG_CW_*×Col-0, or *NahG_CW_*×Laer-0, (data not shown).

### *coi1-*40 and other *coi1* alleles

*coi1-*40 shares certain phenotypes with *coi1-*20 [50]. This allele is also resistant to *Pto*, and induces PR1 strongly upon inoculation. However, *coi1*-20 is male sterile, and the double *coi1-*20 *NahG* does not suppress *NahG* susceptibility [50]. Other alleles like *coi1*-15 and 18 are also male sterile. The mutations in *coi1*-15 and 18 are frameshifts that introduce stop codons [51] while the mutation in *coi1*-20 is unknown. *coi1-*16 is fertile at temperatures below 20°C; however, root growth inhibition and JA-responsive promoter activity are not restored at lower temperatures [52]. Recently, *coi1*-16 was used to recover loci that suppress the ABA signalling pathway, since *coi1*-16 is also hypersensitive to ABA in seed germination [53]. The molecular lesion in *coi1*-16 results in a change of leucine to phenylalanine in the leucine-rich repeat. Lately, two more alleles, *coi1*-21 and *coi1*-22 were described as fertile and impaired in JA signalling [54]. Both alleles have a mutation in the leucine-rich repeat and suppressed the *rar1* phenotype in the resistance triggered by *RPM1* [54]. The mutation in *coi1*-40 induces an amino acid substitution in the F-box domain of the protein [51], a domain in which no previous mutations have been found. In a transgenic line the very same amino acid was changed from glutamic acid to alanine [55]. These *COI1_E22A_* plants did not perceive JA and were male-sterile. The phenotype of lateral roots and trichomes of *COI1_E22A_* was not reported, and seeds for that line are no longer available (Dr. Xie, personal communication), so the only phenotype that we can compare is the fertility of the pollen. Since *coi1*-40 is fertile (Figure 5C), the change to lysine does not inactivate completely the F-Box, as the change to alanine does [55]. We believe that this partial function of the F-Box is responsible of the modular behaviour of *coi1-*40, a theory that is discussed below.

### A modular model for COI1 function

We propose two models to explain the disparity of phenotypes for *coi1*-40. The first one implies that it is a weak allele that retains some function. The difference in phenotypes would be a question of thresholds; some phenotypes are fully functional with the level of signal transduced by *coi1*-40, while others are no longer functional. For example, JA inhibits root growth (Figures 4A and B), and at the same time induces lateral root initiation [35]. If one of the phenotypes (inhibition of root growth) requires a high level of signal to occur, and the other (lateral root initiation) requires a low level threshold, a hypermorphic phenotype in an intermediate *coi1* allele would be observed. Since there are a number of coi1 weak alleles, and none are reported to have hypermorphic phenotypes, this hypothesis is unlikely.

A second and simpler explanation would be a modular or selective function for COI1. In this model, *coi1*-40 would be impaired in some interactions, but others would function near or above wild type levels. Mechanistically, it implies that the mutated F-box is still functional in some interactions, while all other alleles described encode premature stop codons, or changes in the LRRs. However, the expression of JA-induced genes in *coi1*-40 does not provide a strong argument in favour of this hypothesis, although it is worth mentioning that in three out of six genes in mock conditions, the expression levels of the genes in *coi1*-40 are higher than in Col-0 (Figure 6). This fact could be interpreted as an enhanced response to the endogenous levels of JA (explaining the phenotype of lateral roots), but this response is not observed with exogenous MeJA.

Trichome induction is dependent on *COI1* and independent of *MYC2*; therefore there are other components in the JA signal transduction (e.g. *MYBs*, [8]). The same coi1-40 protein that leads to *MYC2* related phenotypes could interact in a stronger fashion with the other components. However, COI1 does not interact directly with MYC2, but the JAZ proteins (Jasmonate-Zim domain, [56]) are the required link. COI1 interacts with ASK1 to form the SCF^COI1^ complex [8] that leads to the degradation of the JAZ proteins [56]. COI1 binds ASK1 through its F-box domain, while it binds to JAZ proteins through its leucine-rich repeat domain. Since the JAZs are repressors, their degradation allows MYC2 and others to promote the response to JA. In this model coi1-40 would interact with ASK1, and this complex would not promote the degradation of the JAZ proteins that are repressing MYC2, but it would degrade other JAZs. Interestingly, a knock out line of *JAZ1* also showed an inhibition of the main root in media supplemented with MeJA and a significant increase in the number of lateral roots [57]. We propose that in the presence of JA, *coi1-*40 degrades some JAZs while stabilizing others, and each one of the JAZs has its own specific interactions.

## Materials and Methods

### Plant Growth and Inoculation

*Arabidopsis thaliana* was sown and grown as described [45]. Plants were grown in controlled environment rooms (CER) with days of 8 h at 21°C, 150 µmol m^−2^ s^−1^ and nights of 16 h at 19°C. For long day experiments, plants were also grown in a CER with the same conditions, except with 16 h of light and 8 h of darkness. The following genotypes were used: *npr1* [58], *ndr1* [59], *sid2* [60], *cpr1* [14], *cpr5* [15], *dnd1* [16], *lsd1* [17], *rpm1* [23], *rps2* [24], *nho1* [61], *ocp3* [26], *coi1*-1 [34], and *jin1* [28]. The treatments, inoculations, and sampling started 30 minutes after the initiation of the artificial day to ensure reproducibility. *Pseudomonas syringae* pv. *tomato* DC3000 (*Pto*) was maintained as described [62]. *Pto* was used with the pVSP61 plasmid containing *avrRpm1* [62], *avrRpt2* [22], or an empty vector. *Pto(cfa ^−^)* [31], *Pseudomonas syringae* pv. *phaseolicola* isolate NPS3121, and *Pseudomonas syringae* pv. *tabaci* were obtained from Dr. Jeff Dangl (UNC, Chapel Hill, NC, USA). The bacteria were grown, inoculated and measured as described [63]. Systemic Acquired Resistance was performed as reported by [64], inoculating leaves with both incompatible and compatible pathogens using a blunt syringe. *Plectosphaerella cucumerina* was provided by Brigitte Mauch-Mani (University of Neuchatel, Switzerland), and used as described [65]. For all the experiments, at least three independent treatments were performed (three independent sets of plants sown and treated on different dates).

### Chemical Treatments

Benzothiadiazole (BTH, CGA 245704), in the form of a commercial product (Bion® 50 WG, a gift from Syngenta Agro S.A., Spain) was prepared in water for each treatment and applied with a household sprayer. The BTH treatments were done as described in [45] and [66]. Briefly, plants were treated with either mock or 350 µM BTH four times during two weeks, starting when the plants were one week old. Then, the fresh weight of each genotype was recorded in both treatments and expressed as percentage of fresh weight of mock-treated plants.

### JA-related phenotypes

For *in vitro* culture, plants were grown in Johnson's media [67] with 1 mM KH_2_PO_4_. When indicated, the plates were supplemented with either 10 or 50 µM MeJA (Duchefa, Haarlem, The Netherlands), depending on the experiment. The length of the roots was measured with ImageJ software (MIH, Bethesda, MD, USA), and the number of lateral roots, with the help of a magnifying glass. Only lateral roots longer than 0.2 mm were counted. When measuring the effect of MeJA on *Pto* growth, MeJA was applied by spray at 100 µM in 0.1% DMSO (SIGMA, St. Louis, MO, USA) and 0.02% Silwet L-77 (Crompton Europe Ltd, Evesham, UK) one day before *Pto* inoculation [68]. Senescence induced by MeJA was measured as described by [36]. For carotenoid measurements, the protocol described by [69] was followed. In order to quantify the amount of seeds produced per plant, eight *coi1-40* and eight wild type plants were selected by molecular marker analysis from an F2 backcross with Col-0. Eight *coi1*-1 plants were also selected from an F2 population segregating for this mutation. Plants were grown in long day conditions, and when the first fruit had matured, the aerial part was covered with a paper bag to avoid loss of seeds. Once the plant had senesced, the seeds were cleaned and weighed. The number of trichomes on the fifth true leaf of 14-day-old plants grown on plates with 10 µM MeJA was determined with the aid of a magnifying glass as described by [38]. The pictures of trichomes were taken with a JSM-5410 scanning electron microscope (JEOL, Tokyo, Japan) in the Electron Microscopy Service (Universidad Politécnica de Valencia, Spain).

### Western Blot

Immunodetection of PR1 protein was carried out as described [19], using an Amersham ECL Plus Western Blotting Detection Reagent (GE HealthCare, Little Chalfont, UK). The second antibody was a 1∶25,000 dilution of Anti-Rabbit IgG HRP Conjugate (Promega, Madison, WI, USA). Chemiluminescent signals were detected using a LA-3000 Luminescent Image Analyzer (Fujifilm Life Science, Stamford, USA). Immunodetection of the large subunit of RuBisCO was accomplished with a 1∶200,000 dilution of a RuBisCO antibody (a gift of Dr. Luis Cañas, IBMCP) and then as mentioned before for the rest of the detection. The amount of signal was quantified with Photoshop (Adobe Photoshop CS4, San Jose, CA, USA).

### Mutagenesis, screening, and mapping

Once the screening conditions were established, seeds of *NahG_CW_* were mutagenized with 0.15% ethyl methanesulfonate (M0880, SIGMA) for 8 hr, and M2 seed collected from ∼100 M1 plants. For the screening, 15-day-old M2 plants were spray inoculated with *Pto* at an OD_600_ of 0.1. One week later, the inoculation was repeated, and the evaluation took place one week after the second inoculation. To confirm the mutants, the M3 of isolated M2 were similarly inoculated, starting at 28 days after germination. Under these conditions, *NahG* plants either die or are severely affected, while wild type plants look unaffected. For mapping, *coi1-*40 was crossed with the ecotype La*er*-0, and, in the segregating F2, plants were selected by the phenotype of the mutant. CAPS [70] and SSLP [71] markers were used from TAIR [72].

*COI1* was sequenced by specific primers (Table S1). *coi1*-40 can be detected by the primers coi1-40F and coi1-40R, (Table S1) followed by digestion with *TaqI* (Fermentas, Madrid, Spain). For the determination of intragenic vs. extragenic mutations, an F1 was obtained between the suppressor and Col-0. If no susceptible plants segregated in 50–100 F2 plants, the molecular lesion was interpreted as being in the *NahG* gene itself, and therefore the suppressor was considered to be intragenic. Conversely, if in the F2 plants appeared that were as susceptible as *NahG_CW_* and as resistant as Col-0, the suppressor was labelled as extragenic.

### RT-qPCR

Total RNA from 10-day-old plants grown on media with or without 50 µM MeJA was extracted with Trizol (Invitrogen, Barcelona, Spain), following the manufacturer's instructions. The details of the RT-qPCR (MIQE data) are provided as Methods S1.

## Supporting Information

Table S1 **Sequencing primers of** ***coi1-40*** **and molecular markers.**
(PDF)

Figure S1 **Proof of concept of the** ***NahG*** **suppressor screen.** Three accessions (Col-0, Ws-1, and La*er*-0), five single mutants (*NahG_CW_* (this work), *cpr1* [14], *cpr5* [15], *lsd1* [17], and *dnd1* [16]) and double mutant combinations with *NahG_CW_* were spray-inoculated with *Pseudomonas syringae* pv. *tomato* isolate DC3000 (*Pto*) at an OD_600_ of 0.1 at 28 days after germination and again one week later. The pictures were taken one week after the second inoculation.
(TIF)

Figure S2 **Characterization of** ***NahG*** **intragenic suppressors.** (A) Resistance and allelelism test of intragenic suppressors. The resistance (R) of M_3_ intragenic suppressor plants, the non-complementation of the intragenic suppressors with Col-0, the resistance (R) evaluation of the F1 and F2, and the allelism test between these suppressors was checked. For this purpose, four-weeks-old plants (the number indicated as “n”) were challenged as in Figure S1. (B) Quantification of growth of *Pto*. Plants were inoculated as in Figure S1, and the growth of *Pto* quantified as described in Methods. Note that with the first 40 mutants, the screen is by no means saturated. The intragenic suppressors form an internal control, since the screen has been sensitive enough to detect 12 reversions to wild type of a single locus. Assuming a Poisson distribution and the extreme scenario that all the extragenic suppressors belong to different complementation groups, the average ratio of alleles per complementation group of 1.38 implies that in the first 40 mutants there would be a maximum of 25% complementation groups not present [73].
(TIF)

Figure S3 **Salicylic acid content of** ***coi1-*** **40.** Both free and total (free plus conjugated) Salicylic Acid is reported for 28 day-old unchallenged plants. Three samples of 100 mg leaves were frozen in liquid nitrogen. Salicylic acid measurements were performed with the biosensor *Acinetobacter sp*. *ADPWH_lux* as described ([74], [75]). The SA levels of Ws-0 are similar to those in Col-0 (data not shown).
(TIF)

Figure S4 **Dry weight of roots growing with and without JA.** The plants were grown as described in Figure 4A, with and without 50 µM MeJA. At 17 days-old, the dry weight of the roots was measured in both conditions, and their ratio (MeJA treated divided by mock treated) expressed as a percentage. The dry weight was determined after drying the roots for 48 h at 65°C. *jin1* and *coi1*-1 mutants were used as controls.
(TIF)

Figure S5 **Mutation in** ***coi1*** **-40 and comparison of COI1 and TIR1 related F-box proteins from Arabidopsis.** Amino acid sequences of COI1, TIR1 and five other TIR1-related F-box proteins from Arabidopsis (AFB) were aligned using CLUSTALW ([76]). Identical residues in all five AFBs and TIR1 are denoted in yellow and the substitution in the amino acid 22 responsible of the *coi1*-40 phenotype is denoted in red (glutamic acid (E) for lysine (K)).
(TIF)

Figure S6 **Details of the trichomes in two** ***coi1*** **alleles.** SEM pictures of leaf epidermal trichomes of the Arabidopsis mutants *coi1*-1 (**A** and **B**) and *coi1*-40 (**C** and **D**). *coi1*-40 trichomes show bigger base cells, wider stem and more papillae along the trichome surface than the *coi1*-1 mutant (scale bar for A and C is 300 µm, scale bar for B and D is 40 µm).
(TIF)

Figure S7 **Analysis of the F1 between** ***coi1*** **-1 and** ***coi1*** **-40.** *coi1*-40, *coi1*-1 and its F1 were tested as described in Figure 4 and 5, for: (**A**) Lateral roots in plates with 50 µM MeJA. At 14 days old, the number of lateral roots longer than 0.2 mm was counted with the help of a magnifying glass. Note that there is a synergistic effect in the F1, with more lateral roots than its parents. (**B**) Trichomes in plates with 10 µM MeJA. When the fifth true leaf emerged, the number of trichomes was counted with the help of a magnifying glass.
(TIF)

Methods S1 **MIQE data of the RT-qPCRs presented.**
(PDF)
