# Supplementary material for: An Allele of Arabidopsis COI1 with Hypo- and Hypermorphic Phenotypes in Plant Growth, Defence and Fertility
Source: PLoS One. 2013 Jan 30;8(1):e55115. doi: 10.1371/journal.pone.0055115 (PMC3559596; doi:10.1371/journal.pone.0055115)
Supplement: Table S1 — Sequencing primers of coi1-40 and molecular markers. (PDF) [file pone.0055115.s001.pdf]

Table S1 – Sequencing primers of *coi1-40* and molecular marker

Primer	Sequence
COI1-40 CDS1 F	GATCTGCGACCTCGATTTCAA
COI1-40 CDS1 R	AGCAAATCCAGCTTTCGGATT
COI1-40 CDS2 F	TTGAATGAGGATATTGGAATGCC
COI1-40 CDS2 R	GAAACCCCAAAACTCGAGACTAAA
coi1-40 F	GATCTGCGACCTCGATTTCAA
coi1-40 R	CGATCTTTCGGGTCAGTTA
C2-12916335 F	AAAAAGCTTCCCGGACTTTC
C2-12916335 R	CAAGTGTATTAGAATCCAAGCAAA
C4-102622254 F	TGAAACAGCGTGTGAAGATTT
C4-102622254 R	AATAACAGTTTGAGAGTTTTAGAGCAA
