# Supplementary figures and images for: An Allele of Arabidopsis COI1 with Hypo- and Hypermorphic Phenotypes in Plant Growth, Defence and Fertility
Source: PLoS One. 2013 Jan 30;8(1):e55115. doi: 10.1371/journal.pone.0055115 (PMC3559596; doi:10.1371/journal.pone.0055115)

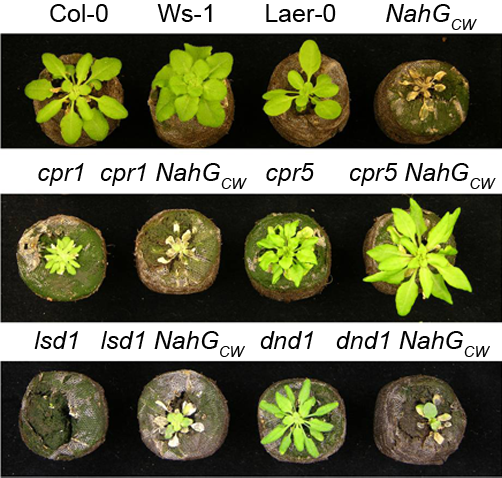

Supplement: Figure S1 — Proof of concept of the NahG suppressor screen. Three accessions (Col-0, Ws-1, and Laer-0), five single mutants (NahGCW (this work), cpr1 [14], cpr5 [15], lsd1 [17], and dnd1 [16]) and double mutant combinations with NahGCW were spray-inoculated with Pseudomonas syringae pv. tomato isolate DC3000 (Pto) at an OD600 of 0.1 at 28 days after germination and again one week later. The pictures were taken one week after the second inoculation. (TIF) [file pone.0055115.s002.tif]

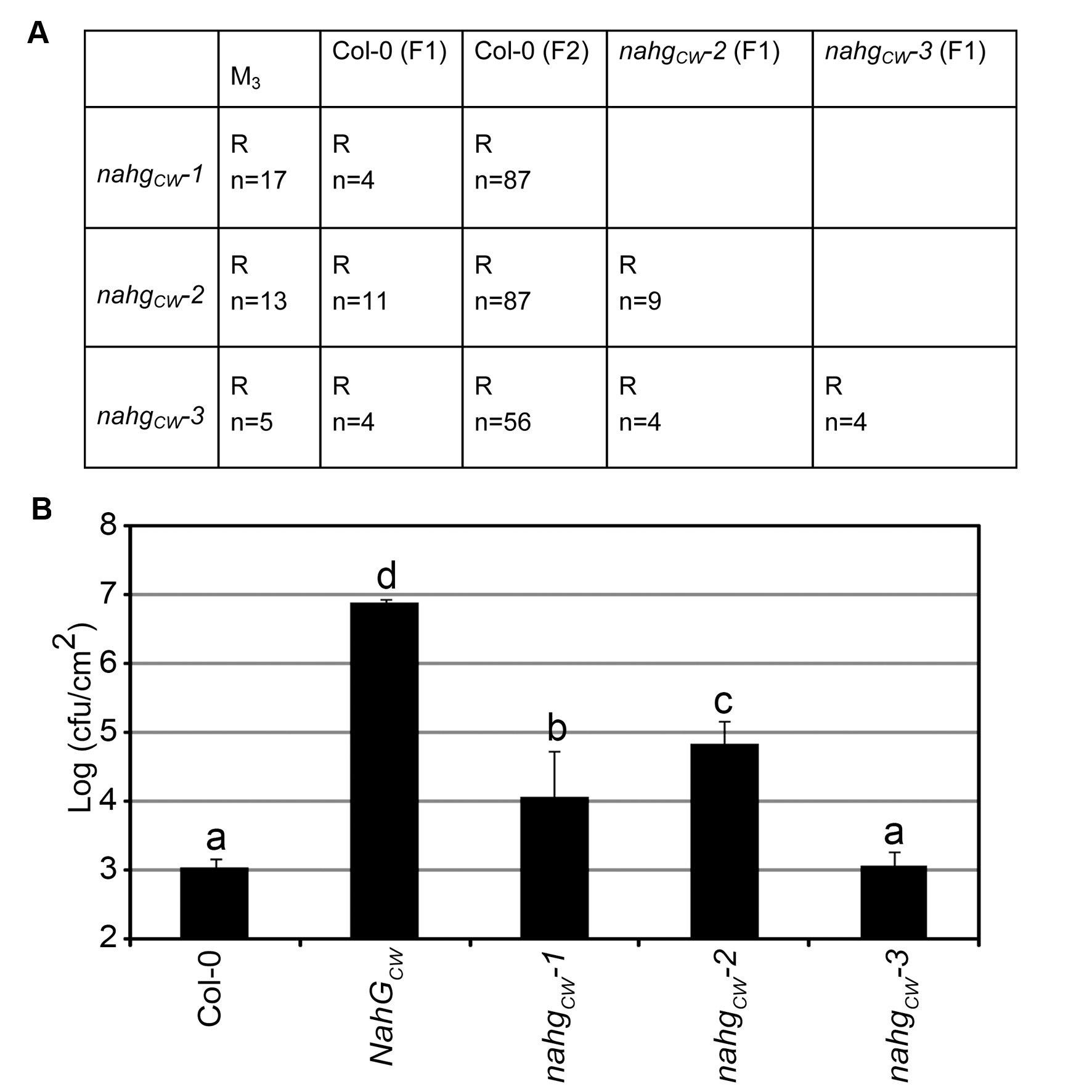

Supplement: Figure S2 — Characterization of NahG intragenic suppressors. (A) Resistance and allelelism test of intragenic suppressors. The resistance (R) of M3 intragenic suppressor plants, the non-complementation of the intragenic suppressors with Col-0, the resistance (R) evaluation of the F1 and F2, and the allelism test between these suppressors was checked. For this purpose, four-weeks-old plants (the number indicated as “n”) were challenged as in Figure S1. (B) Quantification of growth of Pto. Plants were inoculated as in Figure S1, and the growth of Pto quantified as described in Methods. Note that with the first 40 mutants, the screen is by no means saturated. The intragenic suppressors form an internal control, since the screen has been sensitive enough to detect 12 reversions to wild type of a single locus. Assuming a Poisson distribution and the extreme scenario that all the extragenic suppressors belong to different complementation groups, the average ratio of alleles per complementation group of 1.38 implies that in the first 40 mutants there would be a maximum of 25% complementation groups not present [73]. (TIF) [file pone.0055115.s003.tif]

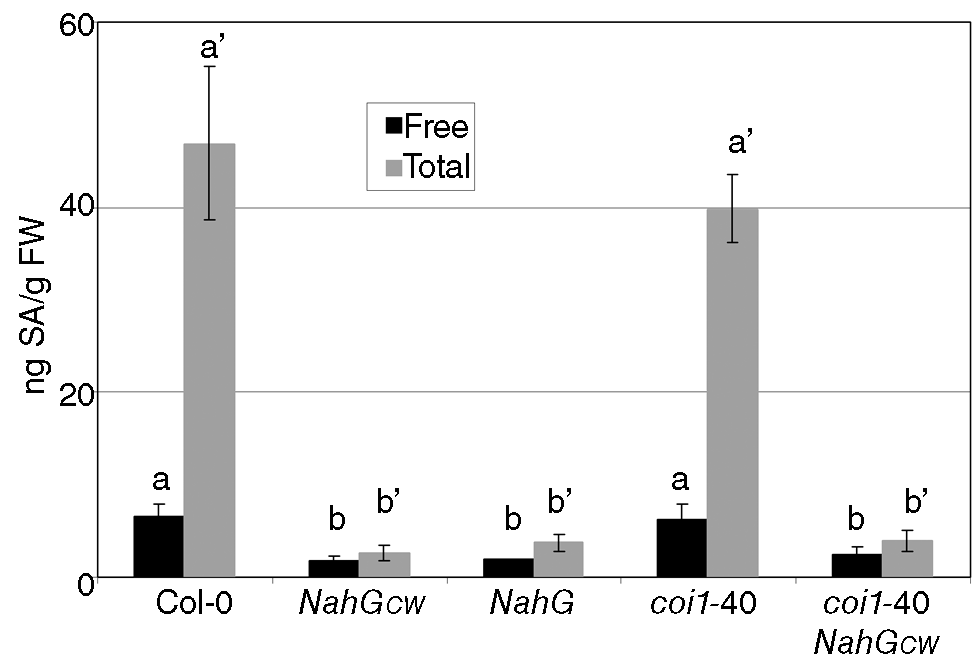

Supplement: Figure S3 — Salicylic acid content of coi1- 40. Both free and total (free plus conjugated) Salicylic Acid is reported for 28 day-old unchallenged plants. Three samples of 100 mg leaves were frozen in liquid nitrogen. Salicylic acid measurements were performed with the biosensor Acinetobacter sp. ADPWH_lux as described ([74], [75]). The SA levels of Ws-0 are similar to those in Col-0 (data not shown). (TIF) [file pone.0055115.s004.tif]

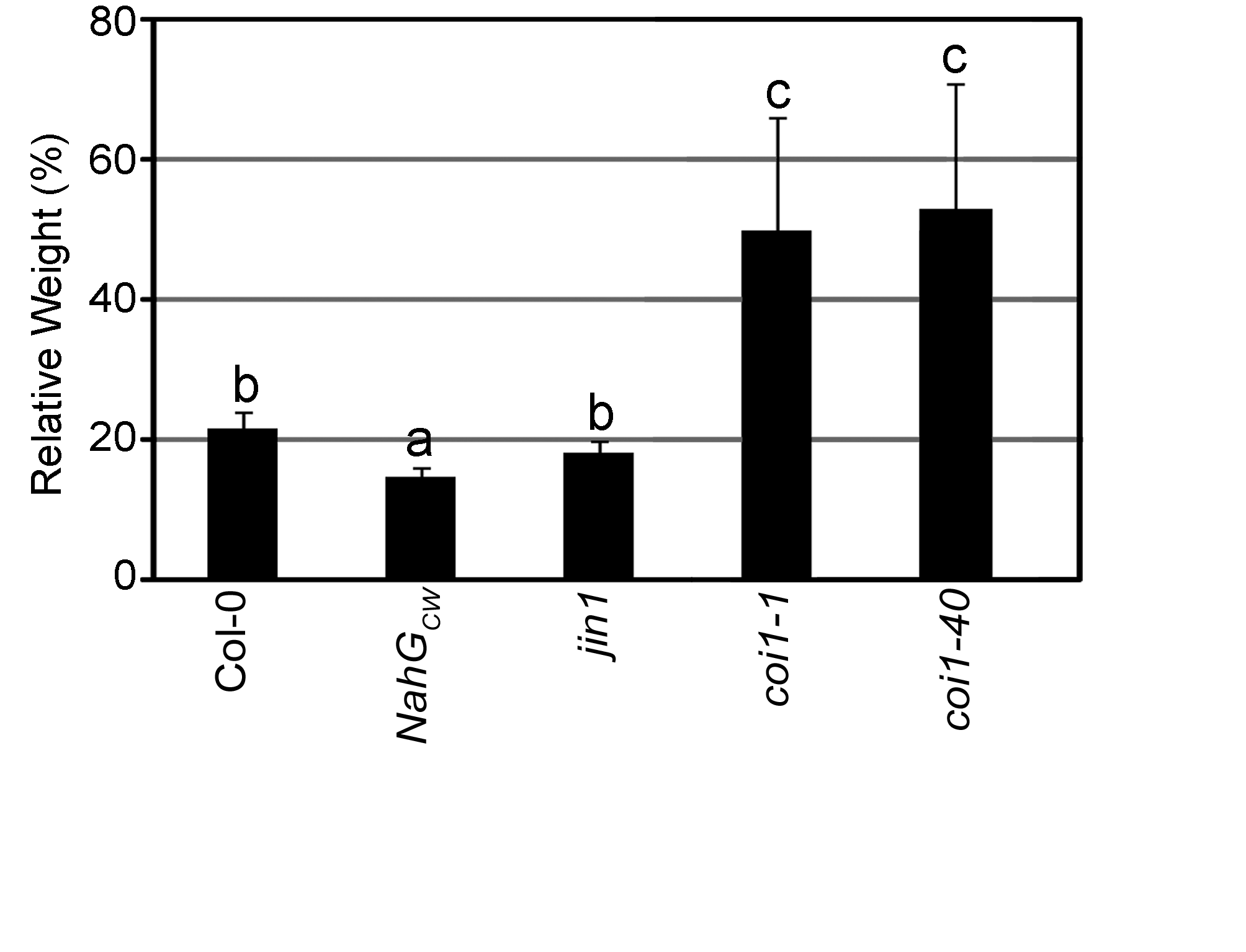

Supplement: Figure S4 — Dry weight of roots growing with and without JA. The plants were grown as described in Figure 4A, with and without 50 µM MeJA. At 17 days-old, the dry weight of the roots was measured in both conditions, and their ratio (MeJA treated divided by mock treated) expressed as a percentage. The dry weight was determined after drying the roots for 48 h at 65°C. jin1 and coi1-1 mutants were used as controls. (TIF) [file pone.0055115.s005.tif]

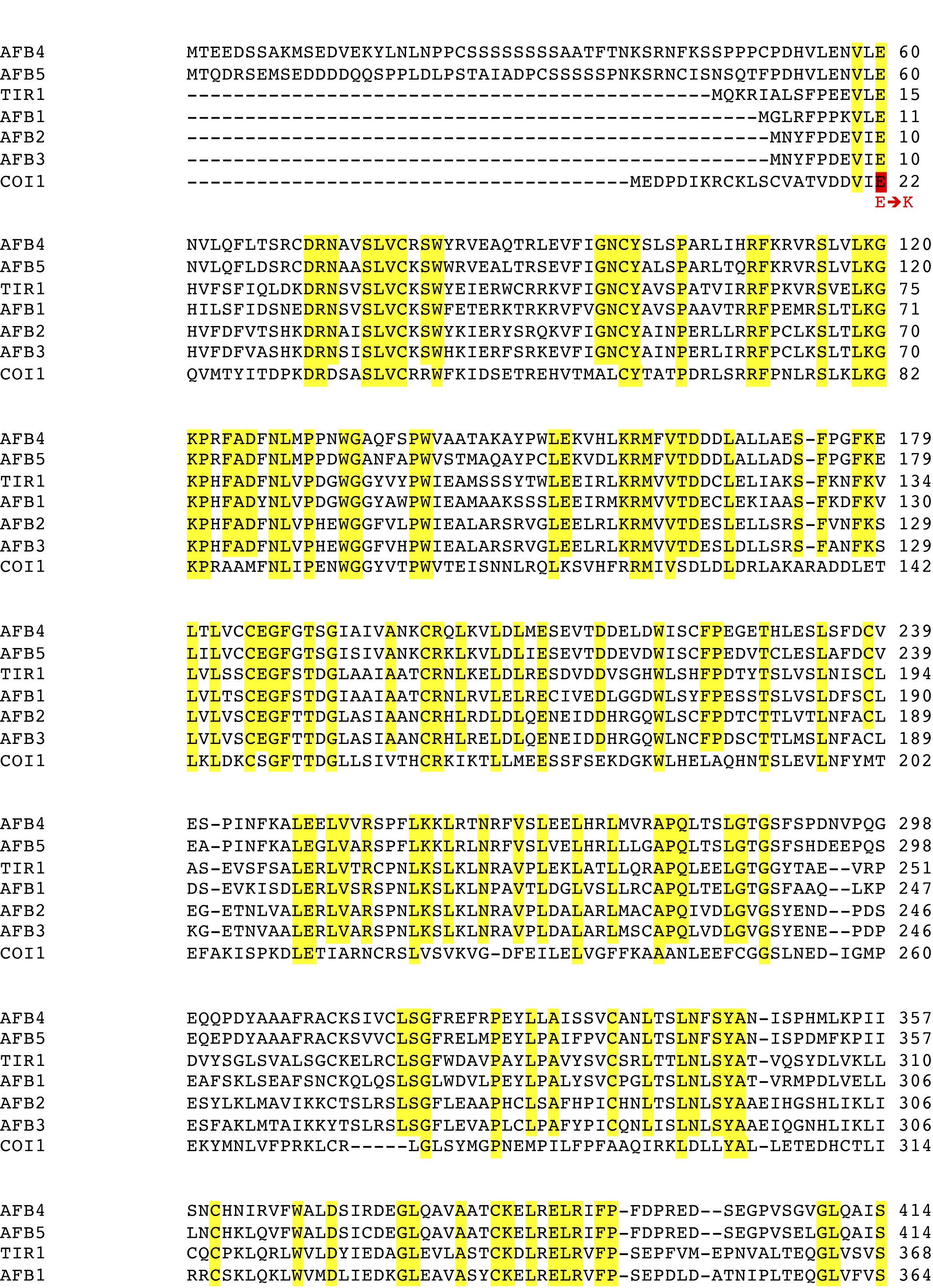

Supplement: Figure S5 — Mutation in coi1 -40 and comparison of COI1 and TIR1 related F-box proteins from Arabidopsis. Amino acid sequences of COI1, TIR1 and five other TIR1-related F-box proteins from Arabidopsis (AFB) were aligned using CLUSTALW ([76]). Identical residues in all five AFBs and TIR1 are denoted in yellow and the substitution in the amino acid 22 responsible of the coi1-40 phenotype is denoted in red (glutamic acid (E) for lysine (K)). (TIF) [file pone.0055115.s006.tif]

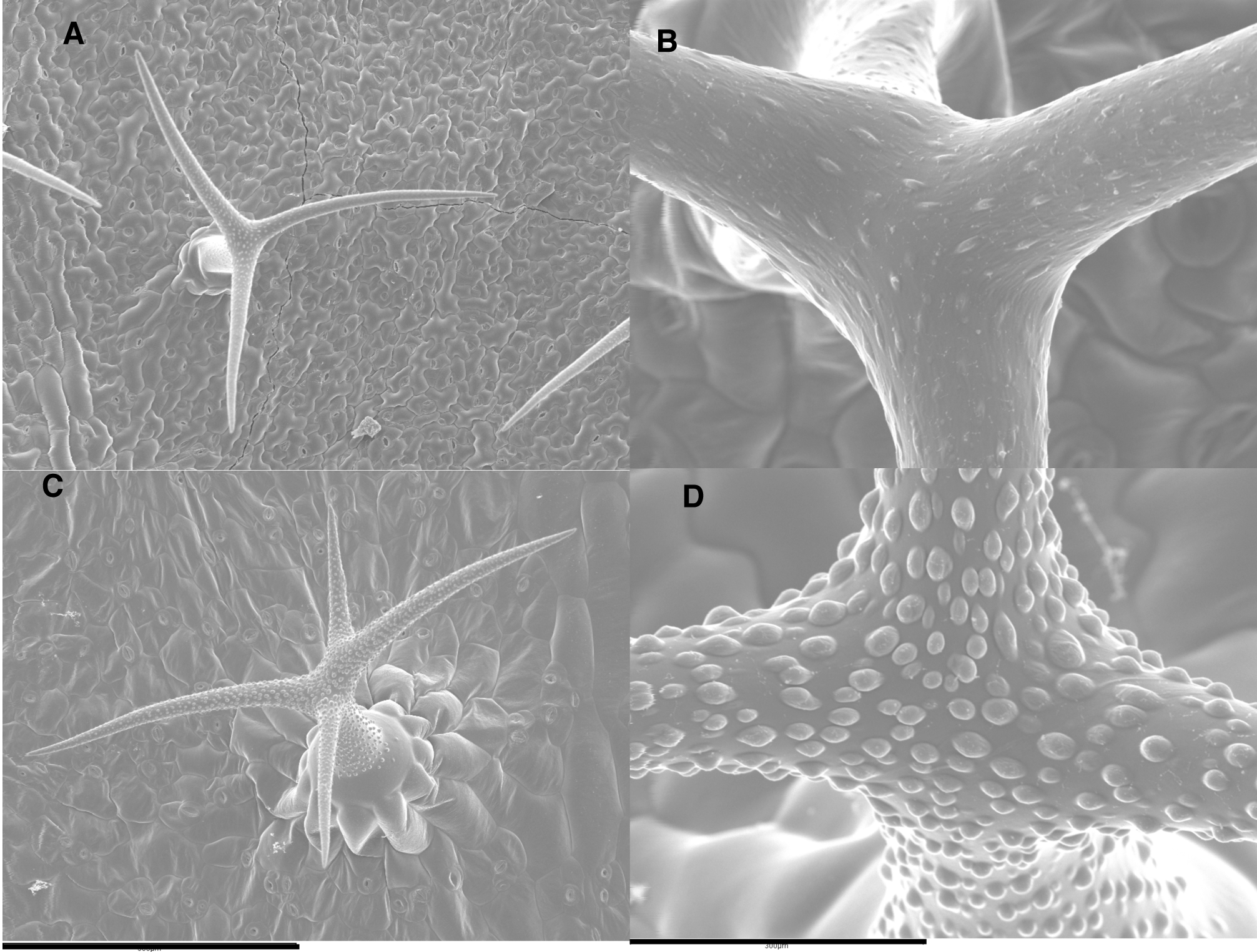

Supplement: Figure S6 — Details of the trichomes in two coi1 alleles. SEM pictures of leaf epidermal trichomes of the Arabidopsis mutants coi1-1 (A and B) and coi1-40 (C and D). coi1-40 trichomes show bigger base cells, wider stem and more papillae along the trichome surface than the coi1-1 mutant (scale bar for A and C is 300 µm, scale bar for B and D is 40 µm). (TIF) [file pone.0055115.s007.tif]

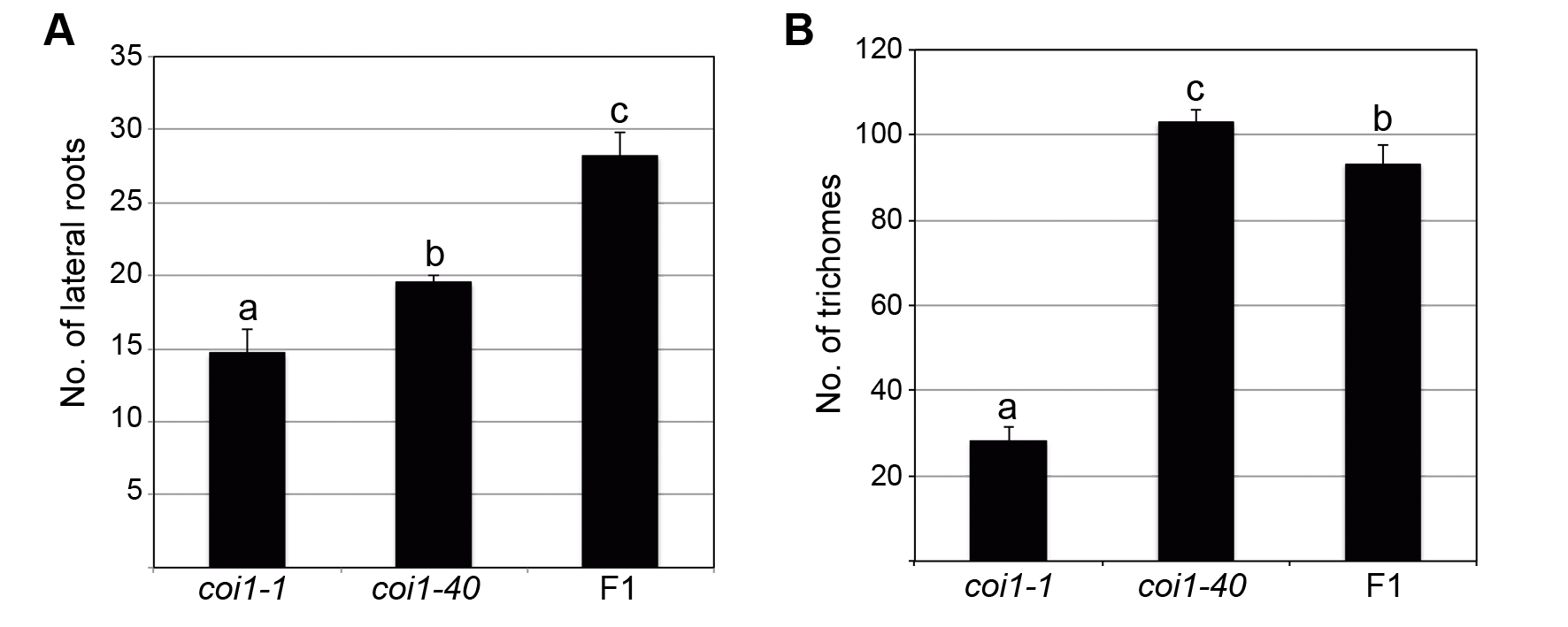

Supplement: Figure S7 — Analysis of the F1 between coi1 -1 and coi1 -40. coi1-40, coi1-1 and its F1 were tested as described in Figure 4 and 5, for: (A) Lateral roots in plates with 50 µM MeJA. At 14 days old, the number of lateral roots longer than 0.2 mm was counted with the help of a magnifying glass. Note that there is a synergistic effect in the F1, with more lateral roots than its parents. (B) Trichomes in plates with 10 µM MeJA. When the fifth true leaf emerged, the number of trichomes was counted with the help of a magnifying glass. (TIF) [file pone.0055115.s008.tif]
