# Supplementary material for: An Allele of Arabidopsis COI1 with Hypo- and Hypermorphic Phenotypes in Plant Growth, Defence and Fertility
Source: PLoS One. 2013 Jan 30;8(1):e55115. doi: 10.1371/journal.pone.0055115 (PMC3559596; doi:10.1371/journal.pone.0055115)
Supplement: Methods S1 — MIQE data of the RT-qPCRs presented. (PDF) [file pone.0055115.s009.pdf]

EXPERIMENTAL DESIGN
Definition of experimental and control groups
Number within each group
Assay carried out by core lab or investigator's lab?
Acknowledgement of authors' contributions
**SAMPLE**
Description
Volume/mass of sample processed
Microdissection or macrodissection
Processing procedure
If frozen - how and how quickly?
If fixed - with what, how quickly?
Sample storage conditions and duration (especially for FFPE samples)

Experimental design is described in materials and methods. Two lab members carried out all the procedures in our lab.

Whole seedlings plants of ten days-old grown *in vitro*, in media supplemented with or without 50 µM of MeJA, were collected in 2mL Eppendorf® tubes and immediately frozen in liquid nitrogen. All samples were stored at -80ºC in a lab freezer during no more than a week before they were processed.

NUCLEIC ACID EXTRACTION
Procedure and/or instrumentation
Name of kit and details of any modifications
Source of additional reagents used
Details of DNase or RNAse treatment
Contamination assessment (DNA or RNA)
Nucleic acid quantification
Instrument and method
Purity (A260/A280)
Yield
RNA integrity method/instrument
RIN/RQI or Cq of 3' and 5' transcripts
Electrophoresis traces
Inhibition testing (Cq dilutions, spike or other)

All samples were frozen-grinded and the RNA was isolated using Trizol®. The protocol of extraction was according to manufacture's procedures sheet. The RNA was then resuspended in 20µL of DEPC ddH_2O. RNA obtained was treated with DNAse I RNAse free (Fermentas), and samples were checked on a 2% agarose gel stained with ethidium bromide. Contamination was assessed by a no RT control in the qPCR reaction.

RNA quantification was performed using a Thermo Nanodrop and 1µL of each sample was analyzed. The A260/280 and the A260/230 ratios were between 1,9 and 2 for all samples. An average of 1500 µg/µL was obtained for all of them.

The RNA integrity was checked on a 2% agarose gel stained with ethidium bromide.

**REVERSE TRANSCRIPTION**
Complete reaction conditions
Amount of RNA and reaction volume
Priming oligonucleotide (if using GSP) and concentration
Reverse transcriptase and concentration
Temperature and time
Manufacturer of reagents and catalogue numbers
Cqs with and without RT
Storage conditions of cDNA

cDNA was synthesized using RevertAid™ Premium First Strand cDNA Synthesis Kit (Fermentas) from 2 µg of RNA. Random hexamers were used. All the procedures were according to the manufacture's recommendations.

For all cDNAs, Cq values were about: ACT: 23.39; ELF: 21.46; PDF1.2: 27.31; VSP1: 24.65; COI1: 25.64; ASA1: 24.64; MYC2: 24.99; PAP1: 29.09; PAP2: 29.84. Without RT no amplification was detected.
All cDNA samples were stored at -80°C.

**qPCR TARGET INFORMATION**
If multiplex, efficiency and LOD of each assay.
Sequence accession number
Location of amplicon
Amplicon length
In silico specificity screen (BLAST, etc)<br>Pseudogenes, retropseudogenes or other homologs?<br>Sequence alignment
Secondary structure analysis of amplicon
Location of each primer by exon or intron (if applicable)
What splice variants are targeted?

Multiplex qPCR was not performed.
The sequence accession numbers are:
ASA1: NM_120655; COI1: NM_129552 ; PAP1: NM_104541; PAP2: NM_105310; MYC2: NM_102998 ;VSP1: NM_122387 ; ACT2: NM_112764; ACT8: NM_103814; EF1α: NM_125432.

Amplicon lengths are: ACT2: 108 bp; ACT8: 107 bp; EF1α: 76 bp MYC2: 76 bp; VSP1: 65 bp; ASA1: 140 bp; COI1: 91 bp; PAP1: 627 bp; PAP2: 448 bp.

Primers were designed using Primer Express ®. Specificity was checked performing BLAST. They were mapped in exons (where possible spanning an intron) or UTR regions. No splice variants were targeted.

qPCR OLIGONUCLEOTIDES
Primer sequences
RTPrimerDB Identification Number
Probe sequences
Location and identity of any modifications
Manufacturer of oligonucleotides
Purification method

Primer	Sequence
PAP1 F[1]	TTGGTTCCTGAAGCGACGAC
PAP1 R[1]	GTCAAAAGCCAAGGTGTCCC
PAP2 F[1]	TTGGTTCCTGAAGCGACGAC
PAP2 R[1]	CAACGTCAAACGCCAAAGTG
VSP1 F[2]	CACTGTCGAGAATCTCAAGGCTG
VSP1 R[2]	CGTTTGGCTTGAGTATGAGATGC
ASA1 F[3]	GTAGAGAAGCTTATGAACATCGA
ASA1 R[3]	GGTGCACCACTAACTGTTCCCAC
MYC2 F[4]	TCATACGACGGTTGCCAGAA
MYC2 R[4]	AGCAACGTTTACAAGCTTTGATTG
COI1 F	CCATTGTACGCTTATCCAAAAGTG
COI1 R	CTGTCAAGGACCTCTAGACCC
ACTIN2/8 F	GGTAACATTGTGCTCAGTGGT
ACTIN2/8 R	AACGACCTTATCTTCATGCTG
EF1α F	TGAGCACGCTCTTCTTGCTTTCA
EF1α R	GGTGGTGGCATCCATCTTGTTACA

All primers were synthetized by Sigma-Aldrich. No modifications were selected. All were fully deprotected and desalted.

qPCR PROTOCOL
Complete reaction conditions
Reaction volume and amount of cDNA/DNA
Primer, (probe), Mg++ and dNTP concentrations
Polymerase identity and concentration
Buffer/kit identity and manufacturer
Exact chemical constitution of the buffer

Additives (SYBR Green I, DMSO, etc.)
Manufacturer of plates/tubes and catalog number
Complete thermocycling parameters
Reaction setup (manual/robotic)
Manufacturer of qPCR instrument

All qPCR reaction had a volume of 20µL with 1µL of cDNA at a concentration of 600ng/µL. Primers concentration was 0.2µM. The buffer used was Power SYBR Green PCR Master Mix (Applied Biosystems).

MicroAmp® Optical 96-Well Reaction Plates were used.

Thermocycling parameters were:
50ºC 2 min
95ºC 10 min

95ºC 15 sec|
60ºC 1 min | 40 cycles

95ºC 10 sec|
60ºC 20 sec| Dissociation step
95ºC 15 sec|

The reaction setup was manual and the qPCR instrument was an ABI PRISM® 7000 Sequence Detection System.

qPCR VALIDATION
Evidence of optimisation (from gradients)
Specificity (gel, sequence, melt, or digest)
For SYBR Green I, Cq of the NTC
Standard curves with slope and y-intercept
PCR efficiency calculated from slope
Confidence interval for PCR efficiency or standard error
r2 of standard curve
Linear dynamic range
Cq variation at lower limit
Confidence intervals throughout range
Evidence for limit of detection
If multiplex, efficiency and LOD of each assay.

The specificity of the amplification products have been confirmed by size estimations on a 1.5% agarose gel and by analyzing their melting curves. Without a template, no Cq could be determined since it never passed the threshold line.

Gene	Efficiency	Slope	Y-Intercept	R
**ASA1**	1.885	0.275	-4.359	0.99
**MYC2**	1.875	0.273	-1.584	0.99
**VSP1**	1.819	0.260	-4.056	0.99
**PAP1**	1.711	0.233	-5.208	0.99
**PAP2**	1.693	0.299	-4.837	0.99
**COI1**	1.829	0.262	-4.303	0.99
**ACT 2/8**	1,903	0,279	-0,877	0.99
**EF1α**	1,777	0,248	-0.183	0.99

DATA ANALYSIS
qPCR analysis program (source, version)
Cq method determination
Outlier identification and disposition
Results of NTCs
Justification of number and choice of reference genes
Description of normalisation method
Number and concordance of biological replicates
Number and stage (RT or qPCR) of technical replicates
Repeatability (intra-assay variation)
Reproducibility (inter-assay variation, %CV)
Power analysis
Statistical methods for result significance
Software (source, version)
Cq or raw data submission using RDML

The results were analyzed using the linregPCR software. The program determines baseline fluorescence and does a baseline subtraction. Then a Window-of-Linearity is set and PCR efficiencies per sample are calculated. With the mean PCR efficiency per amplicon, the Cq value per sample and the fluorescence threshold set to determine the Cq, the starting concentration per sample, expressed in arbitrary fluorescence units, is calculated.

Justification of number and choice of reference genes: these 3 genes have shown robust results in previous experiments in our lab.
Description of normalization method: 3 endogenous reference genes
Number and concordance of biological replicates: 3
Number and stage (RT or qPCR) of technical replicates: each plate had 3 technical replicates per gene and genotype.
Repeatability (intra-assay variation): Results showed an average 0.5 units of variation between technical replicates with a maximum of less than 1 unit.
